# Understanding the Complex Milieu of Epithelial-Mesenchymal Transition in Cancer Metastasis: New Insight Into the Roles of Transcription Factors

**DOI:** 10.3389/fonc.2021.762817

**Published:** 2021-11-18

**Authors:** Sikiru O. Imodoye, Kamoru A. Adedokun, Abdurrasheed Ola Muhammed, Ibrahim O. Bello, Musa A. Muhibi, Taofeeq Oduola, Musiliu A. Oyenike

**Affiliations:** ^1^ Department of Medical Laboratory Science, College of Medicine, University of Lagos, Lagos, Nigeria; ^2^ Department of Oral Pathology, Dental University Hospital, King Saud University Medical City, Riyadh, Saudi Arabia; ^3^ Department of Histopathology, School of Medical Laboratory Science, Usmanu Danfodiyo University, Sokoto, Nigeria; ^4^ Department of Biological Sciences, Southern Illinois University, Edwardsville, IL, United States; ^5^ Department of Medical Laboratory Science, Faculty of Applied Sciences, Edo State University, Uzairue, Nigeria; ^6^ Department of Chemical Pathology, School of Medical Laboratory Sciences, Usmanu Danfodiyo University, Sokoto, Nigeria; ^7^ Department of Medical Laboratory Science, Ladoke Akintola University of Technology, Ogbomosho, Nigeria

**Keywords:** EMT, embryogenesis, ontogenesis, tumour, metastasis, microenvironment, transcription factors

## Abstract

Epithelial-mesenchymal transition (EMT) is a physiological program during which polarised, immobile epithelial cells lose connection with their neighbours and are converted to migratory mesenchymal phenotype. Mechanistically, EMT occurs *via* a series of genetic and cellular events leading to the repression of epithelial-associated markers and upregulation of mesenchymal-associated markers. EMT is very crucial for many biological processes such as embryogenesis and ontogenesis during human development, and again it plays a significant role in wound healing during a programmed replacement of the damaged tissues. However, this process is often hijacked in pathological conditions such as tumour metastasis, which constitutes the most significant drawback in the fight against cancer, accounting for about 90% of cancer-associated mortality globally. Worse still, metastatic tumours are not only challenging to treat with the available conventional radiotherapy and surgical interventions but also resistant to several cytotoxic agents during treatment, owing to their anatomically diffuse localisation in the body system. As the quest to find an effective method of addressing metastasis in cancer intervention heightens, understanding the molecular interplay involving the signalling pathways, downstream effectors, and their interactions with the EMT would be an important requisite while the challenges of metastasis continue to punctuate. Unfortunately, the molecular underpinnings that govern this process remain to be completely illuminated. However, it is becoming increasingly clear that EMT, which initiates every episode of metastasis, significantly requires some master regulators called EMT transcription factors (EMT-TFs). Thus, this review critically examines the roles of TFs as drivers of molecular rewiring that lead to tumour initiation, progression, EMT, metastasis, and colonisation. In addition, it discusses the interaction of various signalling molecules and effector proteins with these factors. It also provides insight into promising therapeutic targets that may inhibit the metastatic process to overcome the limitation of “undruggable” cancer targets in therapeutic design and upturn the current spate of drug resistance. More so, it extends the discussion from the basic understanding of the EMT binary switch model, and ultimately unveiling the E/M cellular plasticity along a phenotypic spectrum *via* multiple trans-differentiations. It wraps up on how this knowledge update shapes the diagnostic and clinical approaches that may demand a potential shift in investigative paradigm using novel technologies such as single-cell analyses to improve overall patient survival.

## Introduction

Embryological evidence of human development shows that the human body is derived from a single cell type, a totipotent cell, from which many other specialised cell types are generated in a bid to expand, differentiate, and grow to accommodate functional diversity. It is based on this understanding that the need for cell transition from one type to another is physiologically justified even though, understanding of similar cell transition in pathological situations, particularly in tumour metastasis, has recently emerged. Importantly, a phenomenon that facilitates such a cellular diversity during tissue/organ development and in adulthood is an epithelial-mesenchymal transition (EMT), characterised by reversible gradual loss of epithelial characteristics and the resultant development of mesenchymal features. As the knowledge of EMT roles in various tissue/organ development surges, three types of EMT have been recognised to occur in biological systems. Each type occurs in response to different biological signals and with different functional consequences (1).

Type 1 represents a physiological process that occurs in implantation, embryo formation, and organ development. Type 2 occurs in association with wound healing, tissue regeneration, and organ fibrosis. This type begins with the initial step of a repair-associated process that generates fibroblasts to regenerate tissue due to trauma and inflammatory damage. In contrast to type 1, type 2 does not continue indefinitely; it stops once the inflammatory process is attenuated (1, 2). In organ fibrosis, however, the EMT process has a propensity for persistent response to recurring inflammatory reactions with the possibility of leading to organ damage. Type 3 predominantly occurs in the neoplastic environment following initial genetic and epigenetic changes affecting oncogenes and tumour suppressor genes that conspire with normal EMT regulatory mechanisms to produce outcomes that are far distinct from the other two types of EMT. The cell phenotypes produced in this EMT type may invade and metastasise to distant organs *via* systemic circulation. In an attempt to decipher the molecular interplay of cell transition mechanisms—both in physiological and pathological states—a lot has been elucidated on the signalling pathways in type 1 and type 2 EMT (2); however, the specific cues that orchestrate type 3 EMT in epithelial carcinoma cells are yet to be completely understood.

In the past few decades, scientists have invested a lot of effort to unravel the pathogenesis of tumour metastasis. This has led to the conviction that a successful tumour spread requires several steps. These include the EMT process, tumour cell invasion, intravasation into the vascular system, transition through the circulatory system, extravasation out of the vasculature, seeding at the premetastatic niche, and, finally, survival and growth at the secondary metastatic site (3, 4). In addition, studies have established that tumour metastasis requires intimate interaction and collaboration between cancer cells and other stromal components of the tumour microenvironment (5), including the inflammatory signals (6), which significantly dictate several aspects of the metastatic cascade.

In this article, we review our current understanding of the multiplex interplays between the master regulators that orchestrate tumour progression in consideration of cellular plasticity along a phenotypic spectrum and beyond the concept of the binary switch model. In particular, we discuss the roles of TFs in EMT programming and potentially attractive therapeutic targets that could help mitigate tumour metastasis. We finally call into attention how this development could be better explored using the state-of-the-art therapeutic design during cancer control interventions.

## EMT and Metastasis

The propensity of carcinoma to populate immediate tissues and migrate to remote organs has long been recognised as a dominant feature of tumour metastasis. This composite phenomenon results when tumour cells disconnect from the initial epithelial layer within the primary malignancies, invade the neighbouring microenvironment, intravasate into the vascular system, and ultimately permeate distant organs (7).

Scientists have proven beyond doubt that the local microenvironment provides necessary signals that determine the fate of the disseminated cells at the peripheral metastatic site, whether to proliferate and revert to a more epithelial phenotype or remain dormant for an extended period (8). In either case, metastasis results. Recent studies have signalled that these events may be supported by the acquisition of EMT features, which is characterised by the loss of apical-lateral polarity (9), even though the complex molecular interplay in this event remains a puzzle. Understanding the molecular mechanism in the EMT program for initiation of tumour invasion and metastasis is key to improved therapeutic interventions.

### Molecular Reprogramming in EMT

Despite the seemingly simple definition, EMT is an extremely complex process, activated by pleiotropic intrinsic and extrinsic factors and finely regulated temporally and spatially (10). It involves several molecular re-engineering that instigates polarised epithelial cells, which ordinarily interact with basement membrane through its basal surface to ultimately gain mesenchymal features. Consequently, loss of apical-basal polarity, enhanced motility, invasiveness, immunosuppression, and increased resistance to cell death and therapy result owing to the acquisition of stem cell-like properties (11). Meanwhile, the hallmark of EMT is the inhibition of E-cadherin. Suppression of E-cadherin results in reduced cell adhesion and imposition of migratory mesenchymal cells. This process is not a snap approach. Even though it was previously believed that the transition of tumour cells from pure epithelial to pure mesenchymal state followed a binary-switch model, the emerging knowledge shows that E→M transition is involved with a complex dynamic process through a phenotypic spectrum. Again, the EMT is a highly conserved phenomenon involving corporations among TFs, effector proteins, and signalling molecules that rely on the activity of TFs as effector molecules.

**Figure 1:**
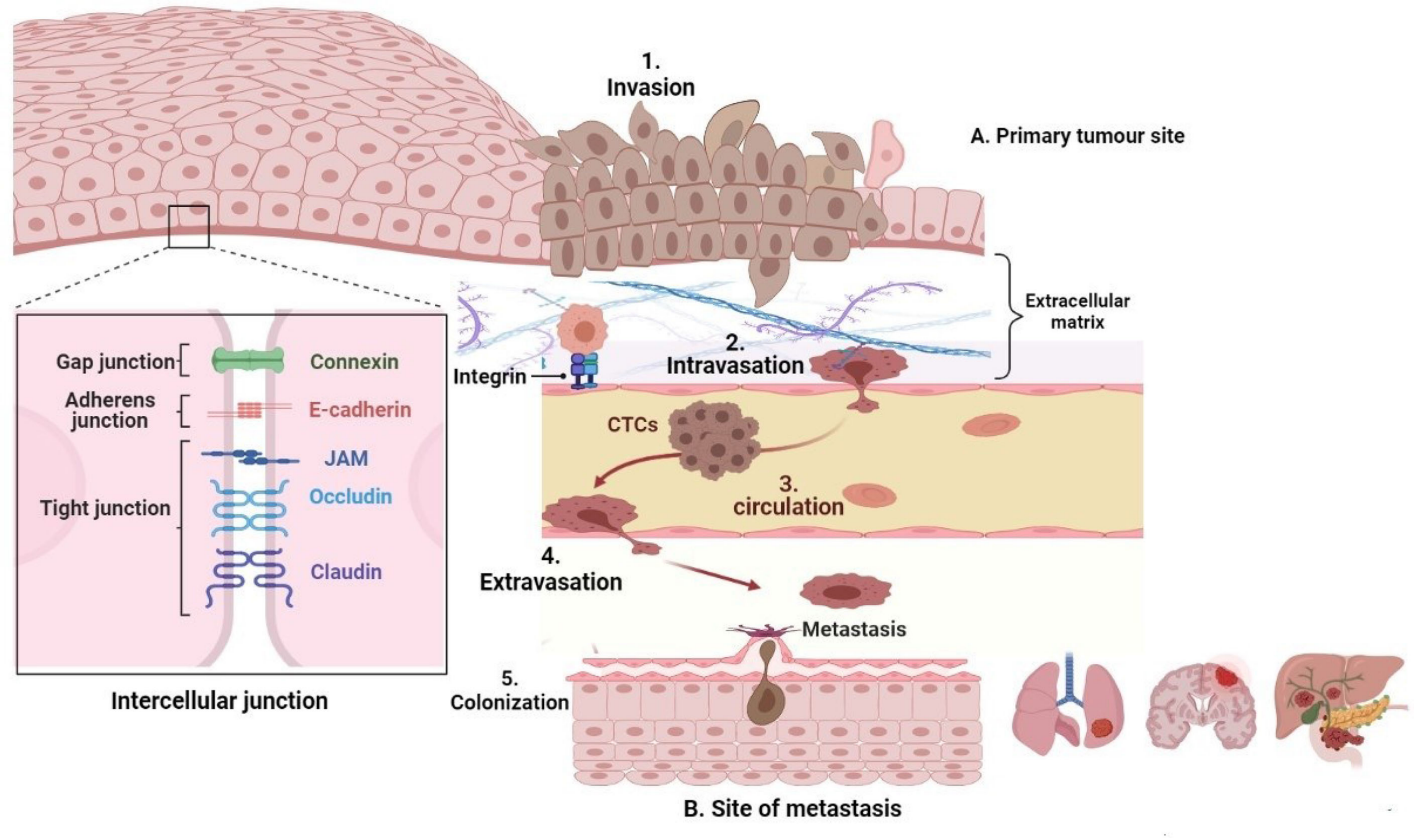
The metastatic cascade. Epithelial tumours may sometimes undergo EMT to generate mesenchymal cells with more motile and invasive properties that penetrate the basal lamina (invasion [1]) and enter the bloodstream or lymphatic system, becoming circulating tumour cells (intravasation [2]), which are transported *via* the systemic circulation (circulation [3]), migrate into distant tissues that have the favourable cellular cues (extravasation [4]). The microenvironmental signals then induce an EMT reversal (also called MET) to establish secondary micrometastases (colonization [5]).

Generally, EMT is characterised by the suppression of epithelial markers particularly involving the intercellular junction protein complex made up of gap junction proteins (such as connexin) adherens junction proteins (particularly, E-cadherin), tight junction proteins (occludin, claudin, and junctional adhesion molecules), cytokeratins, and catenins, as shown in **Figure 1**. Primarily, E-cadherin is the most notable among all epithelial markers for its gatekeeping role to avert EMT by acting in concert with the intercellular adhesion system to maintain cellular polarity, differentiation, migration, and signalling in proliferation pathways. Consequently, loss of E-cadherin promotes the transformation of stationary epithelial cells to a migratory mesenchymal phenotype through a cascade process involving several intermediate E/M phenotypes. Apart from mutation, loss of E-cadherin may also be orchestrated by downregulation due to epigenetic or transcriptional silencing (12, 13). All the same, mesenchymal markers such as vimentin, N-cadherin, fibronectins, smooth muscle actin, and matrix metalloproteases (MMP) also become upregulated and function concurrently with the loss of E-cadherin during the EMT program (14) (**Figure 1**). Altogether, these effector proteins collaborate to induce rearrangement of cellular architecture leading to destabilisation in cellular adhesion, proteolytic degeneration of basement membrane, increased motility (15), and generation of circulating tumour cells (CTCs) (**Figure 1**).

**Figure 2:**
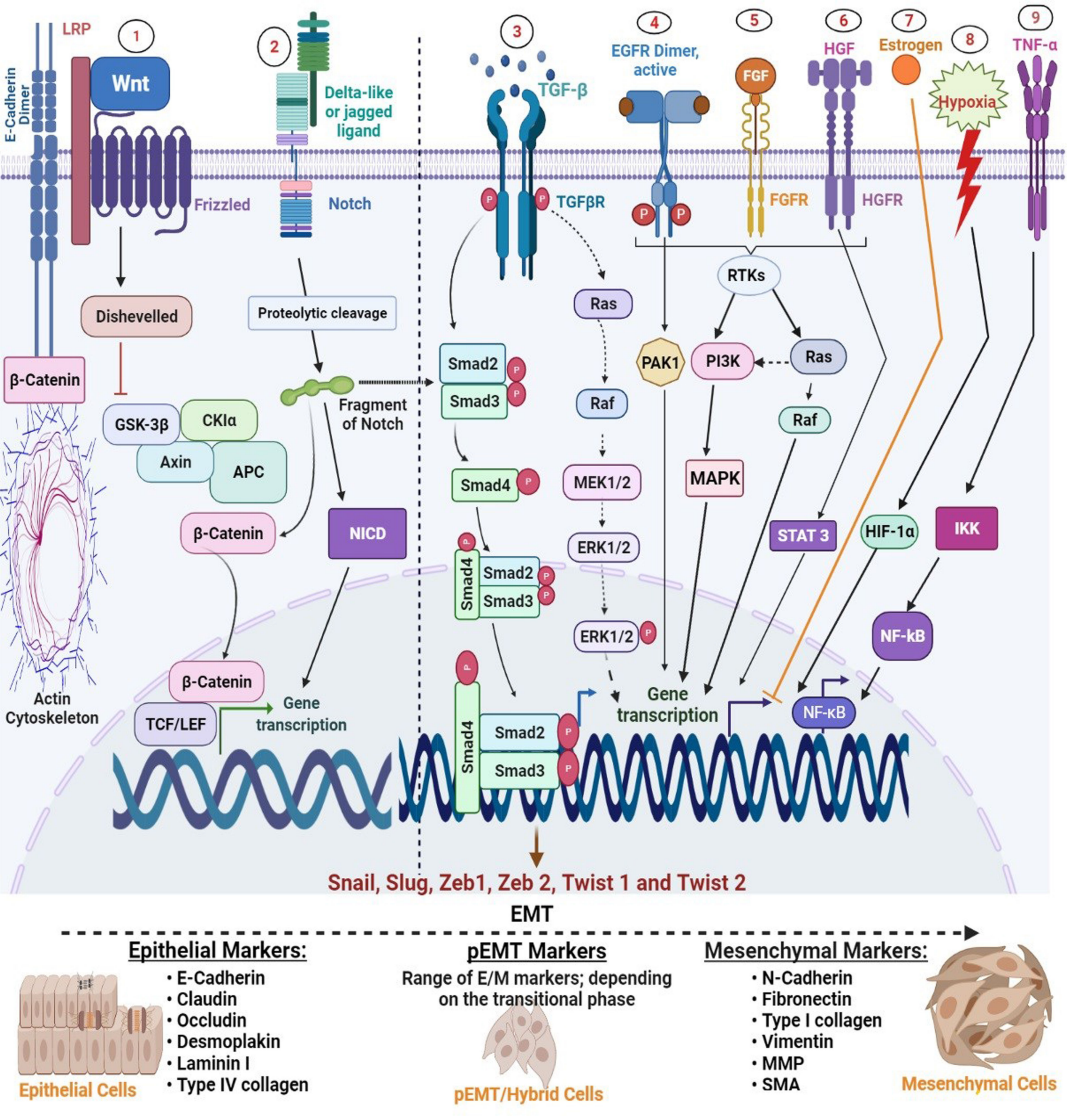
Regulation of major EMT transcription factors by signalling pathways. Epithelial-mesenchymal transition (EMT) is driven by SNAIL, zinc-finger E-box-binding (ZEB), and basic helix-loop-helix (bHLH) transcription factors that act as downstream effectors from several other signalling molecules. 1, Wnt signalling: WNT inhibits glycogen synthase kinase-3β (GSK3β) to stabilize β-catenin, which translocates to the nucleus to recruit the transcription factors; lymphoid enhancer-binding factor 1 (LEF) and T-cell factor (TCF) and promotes expression of SNAIL1 and SNAIL2. 2, Notch signalling: binding of a ligand (such as delta-like or jagged) with Notch receptors initiates the Notch signalling through the proteolytic cleavage by γ-secretase complex to release NICD. NICD then undergoes nuclear translocation to regulate the expression of target genes, including SNAIL, ZEB, and bHLH families. 3, Transforming growth factor-β (TGFβ) promotes EMT by acting at various strata; SMAD-mediated and non-SMAD signalling (i) SMAD-mediated signalling: Activation of the TβRI and TβRII turns on SMAD2 and SMAD3, which then integrate with SMAD4 to form a trimeric SMAD complex. This complex translocates into the nucleus to stimulate the expression of EMT transcription factors (ii) non-SMAD-mediated signalling: TGFβ also activates ERK through the RAS-MAKP pathway. Activated ERK can then stimulate the expression of SNAIL1 and SNAIL2 (unspecified in the image). 4–6, Growth factors (EGF, FGF, and HGF): various growth factors such as EGF, FGF, and PDGF activate receptor tyrosine kinases (RTKs), triggering dimerization and autophosphorylation of the intracellular domain of these receptors, which allows them to activate other downstream signalling molecules, including PI3K, PAK1, and STAT 3 and increase expression of SNAIL1 and SNAIL2. 7, Oestrogen; oestrogen is one of the few molecular candidates that negatively regulate EMT. It does this through direct inhibition of Slug transcription by forming a co-repressor complex consisting of ligand-activated ERα, HDAC1, and nuclear receptor co-repressor (NCoR) that binds to the oestrogen-response elements at the slug promoter sequence and inhibit its expression. 8, Hypoxia: in normoxic conditions, prolyl hydroxylases are activated which causes hydroxylation of HIF-1α and thereby hindering their activities, whereas, hypoxia stabilizes the enzymes, enabling their nuclear translocation and heterodimerization and binding to oversee stimulation of Twist and stabilization of SNAIL1/2. 9, Tumour necrotic factor-alpha (TNF-α): TNF-α signal activates IKB kinase complex (IKK), which in turn phosphorylates NF-kB inhibitor. Consequently, NF-kB becomes active and undergoes nuclear translocation to promote induction of Twist1 expression.

Even though downregulation of E-cadherin has been shown to induce EMT independently in some cancer models (16, 17), a study carried out by Vafaizadeh and her colleagues (18) suggests that active β-catenin is essential for invasion in culture and experimental metastasis model attributed in part to detachment of adherens junctions during early EMT and subsequent degradation. Deducing in tandem with their structural arrangement, the implication of these findings is that β-catenin acts cooperatively with E-cadherin to initiate EMT (**Figure 2**), suggesting that β-catenin may be an important therapeutic target in certain types of cancer.

More so, various coordinated events that occur in EMT are controlled by a set of TFs in a cell-specific and context-dependent manner (19, 20). As illustrated in **Figure 2**, there is vast evidence that these factors are themselves controlled by various signalling pathways and microenvironment signals such as tumour growth factor-beta (TGF-β), epidermal growth factor (EGF), oestrogen, platelet-derived growth factor (PDGF), WNT, hedgehog (SHH), Notch, and integrins (21–26). In addition to induction of EMT by TFs and signalling molecules, certain types of cellular effectors, such as microRNAs, have also been implicated for their roles in stimulating EMT through TFs. In essence, epithelial-mesenchymal transition TFs (EMT-TFs) perform central roles during the EMT program, which is fundamental to various processes in tissue growth and organ development.

**Figure 3:**
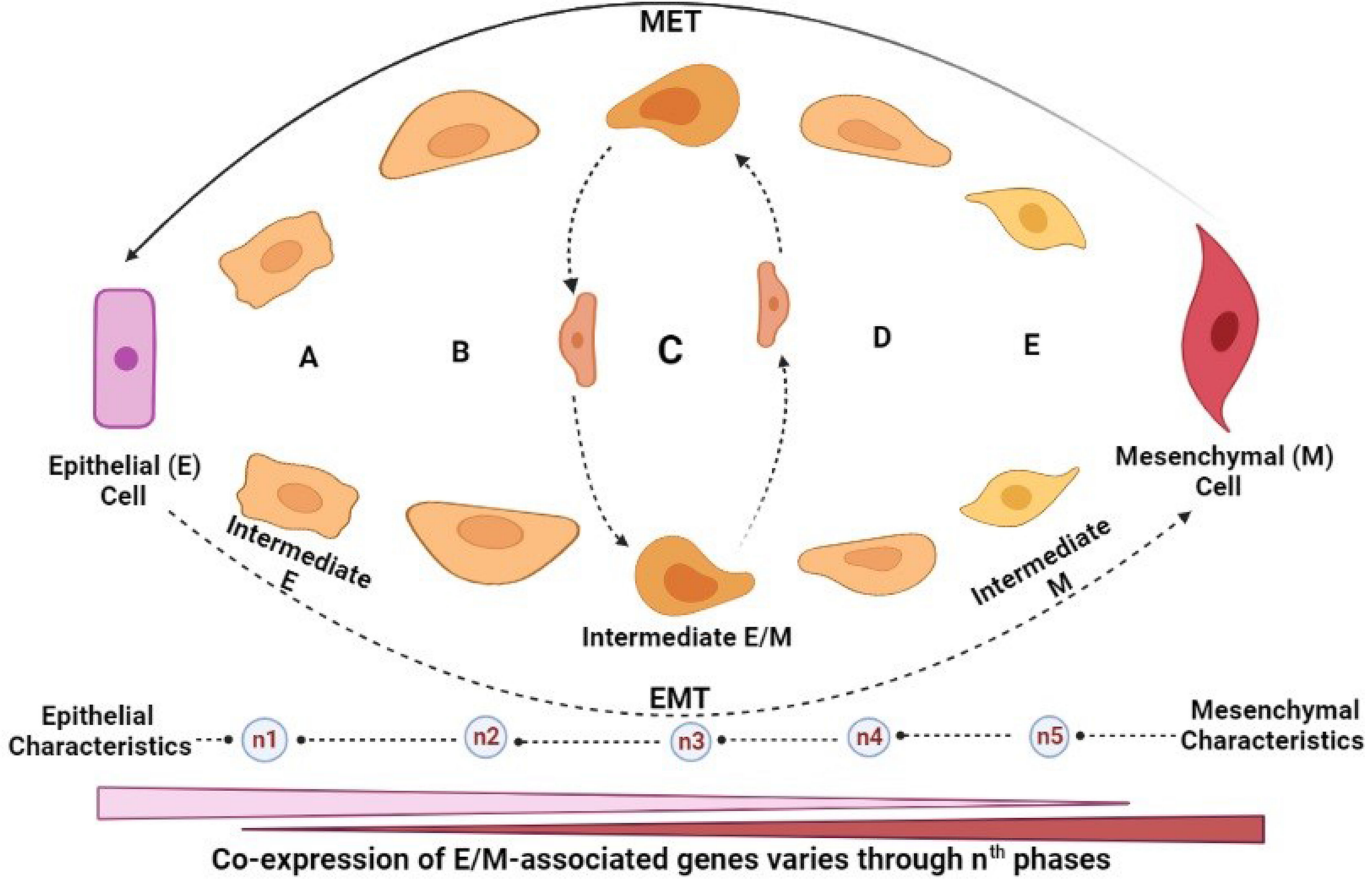
EMT/MET intermediates. **(A–E)** Intermediate cells: hybrid/partial EMT (pEMT) cells. *n*th: The transition phase indicates the number of transitions that may occur before M cells are formed, which is indeterminate but could be dictated by many factors including the tumour microenvironmental cues. Intermediate cells possess dynamic characteristics that are central to increased metastatic potential. Hybrid/pEMT “C”: indicates an unspecific hypothetical stage. The time taken (known as first arrival time (FAT) distribution) for E or M cells to transition to each other, or individual pMET/hybrid cell at the *n*th phase varies depending on (i) epigenetic changes (histone modification or methylation), (ii) presence of anti-EMT/pro-MET factors (such as OVOL 1/2, GRHL-2, ESRP-1/2, and various microRNAs (including miR20 family), among others. Generally, the stability of hybrid E/M is controlled by some factors known as phenotypic stability factors (PSFs) and their metabolic, genomic, and morphologic states. However, as these intermediate cells possess some similar characteristics, they also have different phenotypic behaviours. They possess identical properties such as tumour-initiating potential but are also significantly contrastive, based on (i) cellular plasticity, (ii) degree of invasiveness, and (iii) metastatic potential. Among all the intermediate phenotypes, however, intermediate M possesses the highest migratory potential, the highest degree of invasiveness, most primed to form spindle-shaped phenotype and the highest resistance to anoikis.

Before now, most experimental models have defined EMT as a binary transition of tumour cells between EMT and MET as mentioned above. However, this oversimplification has caused some confusion, as it fails to explain the real phenomena of the current knowledge, which may not address the limitations of modern clinical settings. Several attempts have been made to elucidate the mechanistic process of EMT through various studies and town hall discussions. Recently, during the 2017 and 2019 meetings of The EMT International Association (TEMTIA), the new concept of “epithelial-to-mesenchymal plasticity” (EMP) was introduced (27), thus broadening the traditional definition of EMT to accommodate the newly discovered features such as the partial activation of EMT, and the existence of a continuous spectrum of hybrid EMT/MET phenotype. Presently, the construction of mathematical models and EMT trajectory by single-cell transcriptomics has enhanced our understanding of the existence of multiple intermediate steps with various degrees of E or M states (28, 29) (**Figure 3** **)**. This intermediate E/M phase is characterised by various degrees of molecular markers such as E-cadherin, vimentin, β-catenin, etc., depending on the level of EMT-TF expression or inhibition

Remarkably, emerging studies have suggested that the hybrid E/M status can be further divided into several phases based on the combination of different markers they display, as shown in **Figure 3**. This proposal was supported by a genetically modified skin squamous cell carcinoma model, showing that neoplastic cells commit to spontaneous EMT and that the hybrid E/M status was divided into early and late hybrid E/M states according to the expression patterns of the surface markers CD106, CD61, and CD51 (30). The emerging concept of plasticity of EMT/MET through transitional states is further discussed extensively later in this report.

### Roles of Transcription Factors in EMT Program

The cornerstone of every EMT and its reverse, i.e., mesenchymal-epithelial transition (MET) program, is the transcriptional regulators that control the gene expression required for the cellular transitions. The most widely studied among these factors are zinc-finger proteins (SNAIL), zinc-finger E-box-binding homeobox (ZEB), and Twist family of basic helix-loop-helix (bHLH) transcription factors (TWIST) (31, 32) which are altogether called EMT-TFs (**Table 1**).

**Table 1:** Signalling pathways regulate EMT-TFs.

Pathways	Main EMT-FTs regulated	Changes in expression	Mechanism of modulation	Effects	References
TGF-β signalling	SNAIL1/2, ZEB1/2	Upregulation	TGF activates SMAD complex (SMAD-dependent pathway) and ERK (non-SMAD-dependent pathway), which transcriptionally stimulate the expression of SNAIL1/2 and ZEB1/2	Promotes EMT	(33, 34)
			TGF-β regulates SNAIL by inducing sumoylation at the Lys234 residue of SNAIL, which is critical for its ability to induce an EMT	Promotes EMT	(35)
			TGF-β also activate Notch and Wnt pathways, to promote the expression of SNAIL1/2	Promotes EMT	(33)
NOTCH signalling	SNAIL1/2, ZEB1/2	Upregulation	Direct effect: activation of Notch results in cleavage of the Notch intracellular domain (NICD), which undergoes nuclear translocation, binds to SNAIL promoter, and upregulate the mRNA level of SNAIL1/2 and ZEB1/2	Promotes EMT	(36)
			Indirect effect: indirectly activates β-catenin (Snail1/2, ZEB1/2 and TWIST1/2) to promote and regulate EMT	Promotes EMT	(33)
WNT signalling	SNAIL1/2, TWIST1	Upregulation	Wnt stabilizes β-catenin to recruit lymphoid enhancer-binding factor 1 (LEF) and T-cell factor (TCF), which promotes expression of SNAIL1 and SNAIL 2.	Promotes EMT	(23)
			Recombinant canonical WNT3A induces the expression of TWIST and SNAIL2 and N-cadherin and represses the expression of E-cadherin in HER2 (also known as ERBB2)-expressing breast cancer cells in vitro	Promotes EMT	(37)
Hedgehog (Hh) signalling	SNAIL, ZEB, and TWIST	Upregulation	Direct regulation: binding of Hh ligands activate patched homologs (PTCH1 and PTCH2), resulting in the release of smo proteins and initiate intracellular cascades that later activates Gli family transcription factors, which bind to the promoter region of SNAIL1 to stimulate their expressions	Promotes EMT	(38)
			Indirect regulations: Hh signalling activates TGF and wnt to execute their downstream regulations on SNAIL, ZEB, and TWIST	Promotes EMT	
Tumour necrotic factor-alpha (TNF-α)	TWIST1	Upregulation	TNF-α signal activates the IKB kinase complex (IKK), which in turn phosphorylates NF-kB inhibitor. Consequently, NF-kB becomes active and undergoes nuclear translocation to promote induction of TWIST and SNAIL family members	Promotes EMT	(39)

EMT-TFs are gene expression regulators that direct selective gene expression according to the demand of a cell. Their activities are localized in the nucleus, where they have immediate accessibility to the DNA. A common feature of all EMT-TFs is their physiological roles in embryogenesis and organismal development. However, studies have confirmed their aberrant reappearance in cancer cells during tumour development and progression (40, 41). They are activated in the early events of EMT, and they could hence be said to play cardinal roles in development, fibrosis, and tumour aggressiveness. Sometimes, they control the expression of each other and function synergistically at target genes (42).

The conventional feature of all TFs is the direct/indirect inhibition of E-cadherin expression, resulting in the imposition of mesenchymal state and loss of epithelial cell surface biomarkers to gain effective change in phenotype (4). Barring genetic mutations, the overall effect of EMT-TFs on the degradation of E-cadherin to initiate EMT pathway characterizes a structural transformation in the biological properties of epithelial cells. While it is important to understand the mechanisms involved in phenotypic changes from one cellular architecture to another, the roles of individual EMT-inducing TFs cannot be overemphasised as a functional basis for loss of cellular configuration/polarity in epithelial cells, which in turn influences invasion, cell migration, and resistance to anoikis.

### Snail Transcription Factors

All snail protein members encode transcriptional repressors with a similar structural organisation. The C-terminal domain is exceptionally conserved, containing four- to five-type zinc fingers (C2H2) which oversee sequence-specific binding to the E-box element (5′-CAG GTG-3′) of the target genes (43). The N-terminal in humans contains the evolutionarily conserved SNAG domain, which is important for the binding of various transcriptional corepressor complexes to enforce repression of the target genes (44). The central region of Snail is characterised by a serine-rich domain (SRD) and a nuclear export sequence (NES) that regulate protein stability and subcellular localisation of Snail, respectively.

Snail member proteins are the most widely studied modulators of E-cadherin expression, including Snail, Slug, and Smuc. As opposed to Snail and Slug, the involvement of Smuc in the EMT process of human carcinomas is not yet established (45), according to the available data at present. However, as the paradigm of tumour metastasis keeps evolving, it may be worthwhile to investigate the possible involvement of Smuc in different cancer models.

Also, E-cadherin is the hallmark of EMT, and its suppression is mainly attributed to the functions of Snail1 and Snail2 expressions by binding directly to the E-box of the promoter region and downregulate their expressions (8). EMT-TFs do not only inhibit E-cadherin expression but also downregulate the transcription of other genes encoding the epithelial junction proteins such as claudin (46), ZO-1 (47), both of which serve to stabilise the tight junctions. This implies that any biological process that tends to upregulate Snail expression will encourage EMT. In addition, Snail1 has also been shown to enhance the expressions of matrix degradation enzyme matrix metalloproteinases 9 (MMP9) (48), leading to EMT cascade, as a consequence of proteolytic action of MMP that reshapes the extracellular matrix and deconstructs the epithelial membrane (49).

Snail has long been known as an effector molecule for various signalling pathways that direct their functions. For instance, because Snail is a vital regulator of E-cadherin, scientists have asked whether the effects of the Notch on this process could be mediated through the Notch intracellular domain (NICD) *via* regulation of Snail1. The answer to this is affirmative since Notch has been shown to indirectly promote EMT through regulation of Snail (**Figure 2**). In further agreement with this, Kar et al. (50) reported that activation of Notch-1 promotes EMT *via* the repression of E-cadherin by Slug. Similarly, a study carried out by Niessen and his colleagues showed that activation of the Notch in the context of TGF-β stimulation results in synergistic upregulation of Snail in endothelial cells (51). From the above study, it is clearly understood that TGF-β signalling plays a key role in the pathogenesis of tumour metastasis and thus could function as a potential target in controlling tumour progression.

As indicated earlier, TFs regulate the expression of one another. For instance, when E-cadherin expression is suppressed, the expression of Snail proteins (SNAI1/SNAI2) is amplified by an autoactivation loop due to the inhibition of nuclear factor-κB (NF-κB) (52). Therefore, the self-stimulation loop of Snail is engendered by the downregulation of E-cadherin *via* SNAIL (Snail1/Snail2). This insinuates that Slug is not only a transcriptional repressor but also a transcriptional activator. Intriguingly, Twist1 also binds directly to E-box on the Slug promoter as a transcriptional activator to induce Slug transcription (53). Likewise, Slug is essential for Twist1 to induce EMT. Meanwhile, a knockdown of Slug completely blocks the ability of Twist1 to suppress E-cadherin transcription (54). By implication, Twist1 activity is insufficient to induce cancer cell invasion and distant metastasis in the absence of Slug, making it an attractive therapeutic target.

Apart from EMT modulation, Snail protein members also block the cell cycle, promote cell survival, and inhibit apoptosis, with additional roles in the induction of metastasis and acquisition of cancer stem cell-like characteristics (55, 56). These findings were further supported by separate reports establishing Slug to increase the amount of CD44, a marker of cancer stem cells (CSCs) (57, 58). By implication, the CSC membrane marker could be a potential target for tumour detection, metastatic prevention, selection of drug targets, and other possible intervention strategies.

### Zinc-Finger E-Box Binding Homeobox Transcription Factor

Another important TF, known as zinc-finger E-box binding homeobox, or ZEB, also modulates the transition of epithelial cells to mesenchymal phenotype. Generally, homeobox genes are conserved in plants, fungi and animals. In humans, the ZEB family proteins are made up of two homologous proteins; ZEB1 (δEFI) and ZEB2 (SIP1) which constitute zinc-finger TFs (59). Structurally, they include two ZnF domains, N-terminal ZnF and C-terminal ZnF. The homeodomain consists of three-alpha helices. In this segment, the helix-loop-helix (or helix-turn-helix (HTH)) motif functions as the E-box-binding site and allows the protein to bind to the E-cadherin promoter region (42), and thus regulates the transcription of the adjacent DNA. Generally, the homeobox codes for a specific sequence that functions as part of evolutionarily conserved signalling pathways, which is particularly active in morphogenesis.

Currently, the roles of the proteins of ZEB have been further implicated in tumorigenesis and metastasis as a resultant disruption of morphogenesis. They function through transcriptional inhibition of the E-cadherin gene (CDH1) by binding the promoter site known as a consensus sequence in addition to corepressor recruitment, histone deacetylase process, and chromatin condensation ultimately initiating EMT (60).

In physiological states, however, the proteins of ZEB mainly occur in the heart, CNS, skeletal muscle, and hematopoietic cells to support organ development. Particularly, ZEB1 is commonly found in the thymus during T-lymphocyte development; whereas, ZEB2 is chiefly found in the spleen during B-lymphocyte development (61), suggesting specific functionality and expression. However, evidence of ZEB1 upregulation has been reported in different cancer types such as pancreatic, lung, liver, colon, and breast cancers (62–66). In the same vein, Yilmaz and Christofori (67) demonstrated that increased expression of ZEB1 decreased the response of cancer cells to therapy. These studies predict that ZEB1 could be an important target in anticancer drug resistance.

By interconnection, Snail1 and Twist1 mutually regulate the expression of ZEB1 (68), while the activated ZEB1, in turn, downregulates multiple genes that enforce epithelial characteristics (69), which is the main event in tumour metastasis. In other words, these proteins could be implicated as markers of poor clinical outcomes in patients with a solid tumour, most especially in the metastatic drive. Akin to the Snail family, various signalling molecules modulate ZEB1 and ZEB2 expression. For instance, TGF-β, Wnt/β-catenin, PI3K/Akt, and Ras/Erk signalling induce ZEB1 (70) (**Figure 2**), whereas micro-RNAs negatively regulate the expression.

Furthermore, posttranscriptional phosphorylation of ZEB1 also has the propensity to modulate their expression levels, the functional role that was not immediately understood. Although, recent findings have proposed an inhibitory effect on ZEB1 function. For instance, Llorens et al. (71) reported that phosphorylation of ZEB proteins within C-terminal ZnF inhibits the ZEB binding to DNA and its transcriptional activity. This study shows that ZEB1 phosphorylation could perform many functions particularly in crosslinking the TGF-β signalling with many other pathways involving signalling molecules (cytokines and growth factors) within the cellular microenvironment.

### Basic Helix-Loop-Helix Transcription Factors

In mammals, two bHLH TFs have been identified, Twist1 and Twist2. These two factors share structural similarities and, as such, bind to the same E-box of DNA response elements of the target genes to regulate their expression (72). They are the most characterized indirect transcriptional repressors of the CDH1 promoter, and they play an essential role in embryo formation, wound healing, and tissue fibrosis (73, 74). In adulthood, they are either deficient or expressed at extremely low levels (75), making their molecular underpinning a striking phenomenon for further study, especially in pathogenesis.

Unsurprisingly, scientific studies have shown that Twist proteins are upregulated in carcinogenesis (76, 77) and metastasis (77). Apart from inhibiting the expression of the epithelial marker, Twist1 also increases the expression of mesenchymal markers, including vimentin, N-cadherin, and fibronectin, reducing cellular adhesion and promoting cellular motility (77, 78). In addition, Twist proteins generally promote cancer stem cell phenotype (79), which explains its association with poor prognosis.

By regulation, posttranslational modification of Twist proteins can impact their functions both positively and negatively; for instance, phosphorylation of Twist1 by MAP enhances its stability, promoting invasiveness and EMT in breast cancer cells (80). Contrastingly, IKKβ-mediated phosphorylation promotes the degradation of Twist proteins (81), providing an insight into possible regulation of EMT, which could further prevent tumour cell motility, invasion, and accordingly, cancer metastasis. Understanding the accumulation of Twist protein as a precursor for cancer metastasis, the “brake system” of this molecular purview renders IKKβ a potential novel target for future therapeutic design in the fight against cancer metastasis.

### Roles of Other EMT Transcription Factors in EMT Program

Apart from the TFs that have been studied extensively, previous studies have indicated other nonconventional TFs in the EMT process. For instance, goosecoid (GSC) represses E-cadherin expression indirectly (82) and has been found in various metastatic breast cancer (83). Also, Krüppel-like factor 8 (KLF8) enhances EMT induction through direct suppression of E-cadherin by regulating its promoter (84), resulting in improved motility and altered cell morphology, which has been implicated in breast, ovarian, and gastric cancer cell lines (84–87).

Additionally, another less-reported TF known as paired-related homeobox 1 (PRRX1) also plays important role in tumorigenesis and promotes tumour invasion. PRRX1 with two isoforms—PRRX1a and PRRX1b—exerts distinct functions in EMT. A report from recent studies shows crucial roles of PRRX1 *via* isoform switching during MET, tumour invasion, and metastasis of pancreatic malignancy (88). The study further suggests that PRRX1b isoform stimulates cellular de-differentiation, EMT initiation, and tumour invasiveness while the PRRX1a isoform could induce cell differentiation and the MET (88). Encoded in humans by the *PRRX1* gene, the PRRX1 protein functions primarily act as a transcription coactivator, which enhances the DNA-binding activity of serum response factor that induces many genes by growth and differentiation factors.

Similarly, another superfamily of transcriptional regulators named forkhead box (FOX) proteins is emerging as important regulatory players in various gene expressions associated with cell growth, proliferation, and differentiation. Akin to the common TFs, some FOX proteins are also important drivers in embryonic development and biological shift in cellular phenotypes, which may be linked with maintenance of epithelial polarity *via* E-cadherin function.

In particular, a report in the literature shows that forkhead box C2 (FOXC2) induces EMT by indirect suppression of E-cadherin (89). In effect, FOXC2 plays a crucial role in embryogenesis and angiogenesis, among other physiological processes (90, 91). More importantly, FOXC2 contributes to the metastatic process *via* EMT activation in many cancer growths and developments involving cancers of the breast, prostate, and ovary (92–94). Furthermore, using human tumour cell lines, the investigation also reveals that suppression of FOXC2 by short hairpin RNA (shRNA) in an aggressive metastatic breast cancer model could halt the metastatic tendency (92) suggesting that FOCX2 is not only a promising molecular marker for cancer detection but also a potential therapeutic target. Currently, very few TFs remain “druggable.” Worse still, the existence of many TFs outside the nuclear receptor family poses difficulty in targeting those recognisable TFs with small molecule therapeutics, partly due to paucity of data. Therefore, it might be worthwhile to try out targeting the less-exploitable TFs such as GSC, KLF8, PRRX1, FOCX2, GRHL2, and other poorly investigated similar molecules considering their potential regulatory roles within various signalling pathways.

## Anti-EMT Factors

Despite being two clear-cut cellular states, epithelial and mesenchymal states can trans-differentiate into each other through EMT and its reverse, MET. This fluidity suggests that the regulatory loops among the transcription factors (TFs) involve complex interplays between EMT inducers and EMT suppressors. Several EMT promoters have been identified and elucidated earlier in this study; however, it is also important to discuss the roles of other factors with antimetastatic attributes.

### Proepithelial Factors/Anti-EMT Factors

In contrast to the EMT-inducing transcription factors, molecular mechanisms inhibiting EMT are inadequately characterised as of now. Meanwhile, as scientific efforts are intensifying to decipher the batteries of factors that regulate EMT, there seems to be some cheering news as emerging studies continue to provide evidence of possible spontaneous inhibition of EMT through some special transcription factors. These factors could be referred to as proepithelial factors or anti-EMT factors. Some of these factors include OVO-like transcriptional repressor (OVOL1, OVOL2), grainyhead-like transcription factor 2 (GRHL2), CCAAT-enhancer-binding protein alpha (C/EBPα), oestrogen receptor (ER), and p53. Their major role is to counterbalance the EMT-TFs thereby safeguarding the continued residence of cells in an epithelial state (93).

### OVO-Like Transcriptional Factor

OVOL protein family, including OVOL1 and OVOL2, are the vertebrate homologs of *Drosophila* OVO and have been regarded as critical regulators of cellular transformation both in physiological and diseased states. Even though they are also members of the zinc-finger protein family, they are known as the anti-EMT transcription factors that promote the epithelial status. They are functionally important in the embryogenesis of vertebrates and for the maintenance of an epithelial state and terminal differentiation during tissue homeostasis (94). Mechanistically, OVOL1/2 can suppress EMT by direct inhibition of EMT-TFs, such as ZEB1, ZEB2, and TWIST. In addition, it promotes the reverse of EMT, that is, MET (95, 96), thereby inducing the expression of the cell-cell adhesion molecule called E-cadherin.

However, the extent of the reversal remains a conundrum and requires further investigations since the generation of hybrid EMT will amount to more aggressive malignancy and may portray OVOL proteins as spurious anti-EMT factors. Although the existence of varying hybrid EMT intermediates (as depicted in **Figure 3**) could explain different levels of invasiveness, plasticity, and migratory potential, a deeper understanding of how OVOL1 and OVOL2 promote epithelial differentiation and inhibit EMT in the context of different metastatic models, maybe rewarding to reliably label OVOL factors as useful therapeutic candidates. A report that seems to partially address this concern from Wu and colleagues revealed that OVOL2 thwarts TGF-β signalling and blocks EMT during breast tumour metastasis by repressing SMAD4 expression and interfering with SMAD4 and SMAD2/3 complex formation (97). More recently, Xu et al. proposed that OVOL1 coordinates the suppression of proliferation, invasion, and migration in oral squamous cell carcinoma cells by inhibiting ZEB1 expression *via* direct binding to its promoter (98). This further endorses the OVOL2/ZEB1 feedback loop as the controller of the epithelial-mesenchymal plasticity across several carcinomas.

Recently, mathematical modelling of the feedback circuit between ZEB1 and OVOL2 has revealed that in addition to epithelial and mesenchymal states, cells can acquire one or more hybrid epithelial/mesenchymal states (99). In other words, this has been considered the most plastic and aggressive state (99), hence reinforcing the concern about how beneficial OVOL could be as a novel therapeutic signature for overcoming tumour progression. While OVOL1 and OVOL2 have been described as gatekeepers that prevent mesenchymal trans-differentiation and maintain epithelial identity, their regulation is poorly understood.

OVOL1 or OVOL2 knockdown has been shown to significantly increase the mRNA levels of both vimentin and ZEB1. This led to an extensive functional study of OVOL proteins by Maho et al. (100). The study employed two precancerous conditions, actinic keratosis (AK) and cutaneous squamous cell carcinoma (cSCC) and showed that OVOL1 and OVOL2 were upregulated in AK and significantly downregulated in cSCC. While ZEB1 and vimentin were upregulated in cSCC, most AK cells were negative or faintly express the proteins, suggesting that downregulation of OVOL1/2 and upregulation of ZEB1 and vimentin may be associated with the progression of AK to cSCC. Taken together, this study provides abundant evidence to support the pro-MET features of OVOL factors and the importance of the OVOL2/ZEB1 axis in the maintenance of epithelial status. Thus, the OVOL1/2-ZEB1 axis can form an important axis of regulation of EMT in cancer progression.

### Grainyhead-Like-2 Transcription Factors

The grainyhead-like (GRHL) family of transcription factors consist of three members, GRHL1, GRHL2, and GRHL3, which were first discovered in *Drosophila melanogaster* (101). Out of these, GRHL2 has been widely associated with neoplastic diseases. The roles of GRHL2 in tumour pathogenesis seem to be complex and controversial, varying with cancer type (102). Recently, the signalling pathways between EMT and GRHL2 have attracted considerable attention from researchers. In different cancer models, upregulation of GRHL2 expression has been directly correlated with lower EMT scores; whereas, cancers with mesenchymal features have reduced GRHL2 expression. In cultured human colorectal cancer cells, GRHL2 upregulation promotes epithelial states by reversing the epithelial-like shape from a spindle-like shape and increases E-cadherin, β-catenin, and ZO-1, while vimentin is significantly downregulated (103).

A recent study discovered that TGF-β-induced EMT is inhibited by GRHL2, preventing invasion and migration of gastric cancer. In turn, inhibition of TGF-β signalling pathways increased GRHL2 expression (104). Similarly, GRHL2 significantly inhibits TGF-β-induced, Twist-induced, and spontaneous EMT in breast cancer (105). Further mechanistic studies reveal that GRHL2 is directly inhibited by ZEB1, which in itself, a direct target for repression by GRHL2 (106), suggesting that GRHL2 and ZEB1 form a double-negative regulatory feedback loop in breast cancer cells.

### p53 Tumour Suppressor

The p53 tumour suppressor, also known as the “genome guardian,” plays a dominant role in maintaining genome stability and protects the DNA against mutagenesis (107). p53 plays diverse tissue regulatory roles through several mechanisms. It can be regarded as a transcription factor that acts in response to various stress cues, causing cell cycle arrest, cell ageing, and apoptosis. In addition, p53 plays a vital role in controlling the metabolism and antioxidant status of cells (108). Beyond this, p53 has been associated with anti-EMT roles, by restricting the plasticity of epithelial cells during EMT.

Essentially, p53 indirectly promotes M→E by attenuating EMT-TFs (109) *via* the upregulation of EMT-suppressing miRNAs. Thus, p53 has the potential to avert EMT and the associated stem cell-like phenotype across multiple cancers. Categorically, p53 induces the expression of miR-200c, miR-183, and miR-34, which target ZEB, SNAIL, and TWIST families of transcription factors (110). In other words, apart from maintaining genome integrity, a functioning p53 gene would also be crucial for the maintenance of epithelial integrity.

### CCAAT/Enhancer-Binding Protein Alpha Transcription Factor

The transcription factor CCAAT enhancer-binding protein α (C/EBPa) is a widely expressed basic leucine zipper transcription factor that plays a critical role in cellular differentiation (111). It plays a pivotal role in the regulation of the cell cycle and the expression of several lineage-specific genes (112). Recent findings provided persuasive proof that C/EBPα is an important transcription factor required to sustain epithelial architecture of human mammary cells, preventing epithelial-to-mesenchymal transition and thereby acting as a repressor of breast cancer progression *in vivo* (113). C/EBPα factor can activate and represses several target genes, and its roles in the maintenance of epithelial traits have been attributed to its direct transcriptional activation of epithelial markers, such as CDH1 (E-cadherin) and suppression of EMT-TFs such as ZEB1 (114). This is consistent with previous data on hepatocellular carcinoma, where C/EBPα is shown to be a critical negative regulator of TGF-β-induced EMT, promoting inhibition of N-cadherin and maintenance of E-cadherin expression (114).

Since CEBPA mRNA levels were instantly suppressed upon TGF-β treatment, Ana et al. (113) proposed that SMAD3 may be responsible for the repression of CEBPA transcription. This sounds logical since SMAD3 was found to occupy the CEBPA locus upon TGF-β treatment. Depletion of SMAD3, owing to impaired TGF-β-mediated repression of CEBPA, supports a downstream regulatory role for SMAD3 as a transcriptional repressor of C/EBPα expression during TGF-β-induced EMT.

### Oestrogen Receptor

The oestrogen receptor, especially the alpha class, (ERα) plays a cardinal role in blocking the EMT process (**Figure 2**). For example, metastasis-associated protein 3 (MTA3), directly activated by ERα, downregulates Snail expression (115). Also, ERα directly inhibits Slug transcription by forming a corepressor complex consisting of ligand-activated ERα, histone deacetylase 1 (HDAC1), and nuclear receptor corepressor (NCoR) that binds to the oestrogen-response elements at the Slug promoter sequence (115, 116).

Therefore, the absence of ER or MTA3 results in abnormal expression of Snail and Slug diminishing the ER activity to promote metastasis, chemoresistance, and recurrence after treatment (117, 118). These findings justify why ER is regarded as a marker of poor clinical outcomes and an important therapeutic target in breast cancer (119). Thus, through many significant molecular mediations in connection with tumorigenesis, EMT, and metastatic cascade, it might be possible to develop a novel drug targeting the associated ER-signalling components in women breast cancer intervention.

### Regulation of TFs by Signalling Pathways

As stated earlier, EMT-TFs are regulated by various intracellular signalling networks (**Figure 2**). It starts with the extracellular molecules binding to its particular membrane receptor to initiate intracellular signal transduction. The transforming growth factor, wnt, oestrogen, fibroblast growth factor (FGF), PDGF, and the Jagged family bind to TGF-β receptor (TGFβR), Frizzled, oestrogen receptor (ER), fibroblast growth factor receptor (FGFR), platelet-derived growth factor receptor (PDGF-R), and Notch, respectively (120–122). The ligand-bound receptors transduce intracellular signals *via* the pathways, such as MAPK, phosphatidylinositol 3-kinase (PI3K)/protein kinase B (Akt), nuclear factor-κB (NF-kB), β-catenin, or the Smad signalling, which regulate the expression and stability of EMT-TFs (121) (**Figure 2**).

Previous findings have shown that these networks of signalling interface at various strata and numerous feedback activation and inhibition mechanisms have been illustrated in different EMT contexts, with the possibility of showing overlapping and context-specific results (123, 124). This highlights the importance of delineating the induction of these pathways in various subpopulations of cancer cells following EMT activation to understand possible therapeutic targets in various human carcinomas.

Notably, the dominant roles of TGF-β in EMT must be acknowledged. It is a well-elucidated molecule that induces EMT in various cellular contexts, cancer inclusive. The functions of TGF-β in cancer vary from one biological setting to another, deciphering its dual relevance in tumour pathogenesis, which has been called the “TGF-β paradox” (125). In the early tumour stage, TGF-β supports apoptosis and inhibits the proliferation of tumour cells. Conversely, it plays a tumour-promoting role in the later stage by stimulating EMT, genomic instability, angiogenesis, cell motility, and metastasis.

Following successful binding to its receptor (TGF-βR), it activates several signalling cascades, including Ras-MAPK and SMAD-dependent signalling. Activation of the RAS-MAPK induces expression of Snail1 and Snail2, ultimately leading to the repression of E-cadherin (103). TGF-β can also initiate gene expression through activation of the Notch, and Wnt pathways, which then lead to activation of Snail1 and Snail2, expression of mesenchymal markers, and EMT induction *via* degradation of intracellular catenins (33) (**Figure 2**).

In addition to ligand-receptor interactions, environmental signals such as oxygen reduction in the cellular environment can as well directly activate TGF-β through hypoxia-inducible factor 1 alpha (HIF-1α) (126) which in turn leads to gene transcription. More so, HIF-1α can initiate epigenetic modulation of EMT by targeting the transcription of histone deacetylase 3 genes (HDAC3), cooperating with Snail and ZEB expressions to suppress epithelial expression (127), with the imposition of mesenchymal characteristics.

Furthermore, there is compelling evidence in recent years to support the significant role of Notch in the EMT process (128–130). This is not surprising because the pathway is the nexus of an adaptable signalling network that controls various cellular mechanisms in different biological contexts—determined by the tissue microenvironment (131, 132). Notch signalling explores two discrete channels to synergistically regulate the expression of EMT-TFs (133). Firstly, direct activation of TFs by mobilising the Notch intracellular domain (NICD) to their promoter and secondly, by potentiating HIF-1α recruitment to the lysyl oxidase (LOX) promoter to aid the hypoxia-induced upregulation of LOX, which stabilises Snail and safeguarding it from protein degradation (134). Importantly, the signals generated from this pathway usually stimulate transcriptional repressors (Snail1/2, ZEB1/2, and TWIST1/2) to promote and regulate EMT.

Fortunately, studies have proven that inhibition of Notch signalling by the small interfering RNA (*siRNA*) system promotes reversal of the EMT phenotype, resulting in the MET by inhibiting ZEB1, Slug, Snail, and NF-κB (135). These data present molecular evidence connecting Notch signalling with neoplastic drug resistance validated by EMT phenotype. This suggests that inactivation of Notch signalling with a novel targeted therapeutic approach could be conceived to subdue chemoresistance toward preventing tumour progression, for which present traditional therapeutic strategies are grossly dissatisfying.

Interestingly, Snail could also regulate the Notch expression. Kuphal et al. (136), for example, found downregulation of Notch-4 by antisense Snail cDNA transfection of melanoma cells, and the results are convincing in support of the complex crosstalk between Notch and Snail during the acquisition of EMT. These findings indicate the complex contribution of different TGF-β to EMT in carcinoma cells and, therefore, represent an intimidating obstacle in formulating a therapeutic model to control the EMT in human tumours.

### Regulation of EMT by MicroRNAs

MicroRNAs (miRNAs) are a small class of noncoding RNAs that modulate gene expression by RNA silencing, translational suppression, and mRNA degradation (137). They are involved in many biological processes involving cell differentiation, proliferation, cell death, and tumour invasion (138). Another report shows that miR-200 and miR-205 members can mediate EMT suppression by direct inhibition of ZEB1 and ZEB2, thus enhancing the tumour sensitivity to therapeutic interventions (139). In addition, the expression of miR-204/miR-29b demonstrates the blockage of metastasis and cancer invasion considerably in gastric cancer cell lines *via* Snail1-induced EMT suppression (140). Unsurprisingly, however, the low expression level of the miR-200 family in breast cancer enhances ZEB1/ZEB2 transcriptions, inducing TGF-β/BMP signalling to sustain EMT (141). Also, Kim et al. (110) reported that p53 negatively modulates EMT in cancer of the liver upregulating the miR-200 and miR-192 expression by targeting inhibition of ZEB1/ZEB2 expression.

Activation of p53 targets many microRNAs—miR-200c, miR-183, and miR-34—by inducing their expressions. This process, in turn, downregulates some EMT-TFs such as ZEB and Snail. Consequently, the chain reaction in the signalling cascade directly results in EMT suppression and thus MET activation. In essence, induction of TGF-β, mutations, or epigenetic silencing which may cause loss of p53 could ultimately stimulate the EMT process (142).

## Hybrid Tumour Cell Subpopulations: Plasticity Through EMT

As mentioned earlier in this review, EMT was traditionally considered a binary model and that any intermediate state is just a transient snapshot acquired during the EMT process (143). However, our understanding has recently evolved through a plethora of scientific studies, revealing that cancer cells can acquire metastable intermediate transition, with a concoction of cells displaying the features of E or M phenotype or both (E/M) at the molecular and/or morphological level. While several authors have referred to these intermediary states as partial/hybrid EMT (pEMT) (144), some set of scholars (145) have consistently used “quasi-mesenchymal cells” because those cells express *CDH1* gene (which encodes E-cadherin) at the transcript level without displaying E-cadherin at the cell surface (146), in addition to stem-like features and coexpression of certain epithelial and mesenchymal genes.

By studying the plasticity of EMT/MET through transitional states, investigations show a range of pEMT intermediates (30). Indeed, EMT is beyond the binary state; that EMT trans-differentiation phases exist as a spectrum of various intermediate hybrid states raise a big concern for cancer cell stemness and failure in many therapeutics. Although, there is a strong evidence to support that hybrid EMT/pEMT state is associated with increased metastatic tendency, hitherto, it is not understood whether each pEMT intermediate subpopulations have different cellular properties that may dictate their relative responses to treatment with immunotherapy, radiotherapy, and/or chemotherapy. Also, the precise mechanism that drives each transitional state through many intermediate phases is yet unknown. More so, the actual number of phases (here termed “n”, representing a definite number of transitional phases; **Figure 3**) each cancer phenotype undergoes from pure epithelial to mesenchymal state is still beyond the current understanding of EMT. By implication, tumours with pEMT may exhibit increased intratumour heterogeneity.

Although, all EMT-induced states possess aggressive features, those carcinoma cells in the pEMT state have demonstrated greater tumour-initiating and apoptotic potential as well as therapeutic resistance than purely epithelial or mesenchymal cells (147). Similar observations were made in a mouse model of prostate cancer, where both hybrid epithelial/mesenchymal and fully mesenchymal carcinoma cells were shown to initiate primary tumours, but the fully mesenchymal cells were unable to generate macroscopic metastases, while the hybrid cells were capable of doing so (148). Corroborating this finding, another study by Cornelia et al. (149) employed expression vectors and gene knockout models to establish that malignant cells that exist in the hybrid E/M state have highly tumorigenic features but lack plasticity. Instead, they were locked in the E/M state and thereby unable to transit into more E or more M states spontaneously, suggesting that residence in a hybrid E/M state is enough for the preservation of stem cell properties and the associated stemness is thus exhibited regardless of phenotypic plasticity.

More also, cells in pEMT state exhibit loss of apical-basal polarity and have better motility, while retaining adhesive features with the nearby cells and acquiring mesenchymal-like characteristics (144); hence, they can form cell clusters and move together. If these cell bundles are lucky to successfully emigrate to the bloodstream intact, they form clusters of CTCs that can migrate collectively.

Interestingly, hybrid cells coexpressing epithelial and mesenchymal markers have been detected among CTC clusters in the bloodstream of patients with different tumour types, including breast, lung, colon, and prostate cancer (150), thereby complicating our quest to understand the composite milieu of EMT spectrum. Tayoun and his colleagues reported that clustered CTCs have advanced plasticity, possess CSC characteristics and the ability to initiate tumours and form new lesions and are fatal in tumour patients (151). While a few factors that promote CTC cluster formation have been identified (152), the key mechanisms that allow CTC clusters to survive in the vascular system and allow them to metastasise effortlessly than freely circulating CTCs remain contestable.

However, it is convincing to predict that malignant cells that occupy pEMT locus along the EMT spectrum may be the most suitable for metastasis because CTCs that migrate collectively rather than singly may offer superior resistance against the onslaught of the protective machinery within the blood circulations, conforming with the axiom “united we stand, divided we fall!” Interestingly, an earlier study demonstrated that CTC clusters contribute to 50% of the total cells at the secondary metastatic niche despite only constituting 3% of the total CTCs (153). Reinforcing this discovery, Joosse et al. predicted that CTC aggregates have a high probability of being trapped in narrow blood vessels, hence facilitating extravasation (154).

In essence, the fact that neoplastic cells that occupy the pEMT state circulate in clusters, with a greater tendency to become CSC (155), make them behave more aggressively than their corresponding mesenchymal or epithelial counterparts. The mechanism by which pEMT regulates stemness in squamous cell carcinoma (SCC) is still largely unknown, although a study has proposed that clustered CTCs display binding sites for stemness-associated factors such as OCT4, NANOG, and SOX2 are more hypomethylated (155).

## Future Perspective

There is overwhelming evidence connecting EMT with tumour progression and metastasis and sometimes drug resistance, with transcription factors as the key players. Currently, emerging knowledge of cellular plasticity and the continuous spectrum of EMT transitional phases in metastasis establish the presence of multiple tumour cell subpopulations. Importantly, the translational significance of the interrelated events poses huge implications in the orbit of cancer treatment and other important intervention strategies. Here, we bring to the limelight some important approaches to circumvent the drawbacks facing the current cancer management, and further, provide how advancing the understanding of EMT transition beyond the binary model could shape the diagnostic and therapeutic strategies with a novel approach, as follows:

- Reversing the EMT to its opposite state, i.e., MET.
- Inhibiting the EMT initiation.
- Unveiling the prospect of single-cell analysis in EMT.

## Reversing the EMT to Its Opposite State (MET)

Reversing the EMT is a potential therapeutic approach involving a forced stimulation of MET, which in principle should be an exquisite way to prevent EMT [121]. However, the molecular basis of this process is only beginning to gain recognition in recent time compared with the EMT pathway, and therefore it may take a more extended period for a rational design of modulators of this direction to be explored. Mechanistically speaking, interfering EMT transition is crucial to the prevention of metastasis. Importantly, to achieve EMT→MET reversion, tumour staging may be a central point to consider. For instance, at early metastatic stages, Twist1 contributes to tumour invasion and vascular intravasation through repression of E-cadherin expression. Interestingly, however, epithelial cells that predominate metastatic lesions suggest that EMT→MET reversal is also fundamental to circulating tumour cells (CTCs) in colonising a new metastatic niche at distal organs, attributable to loss of Twist1 signal—the same signal that orchestrates tumour invasion and vascular intravasation at early metastatic stages. Without mincing words, the molecular mechanisms that dictate on-and-off switches of EMT-TFs as observable in Twist1 signal at different metastatic phases have received low attention. Thus, the connecting pathway involving Twist1 expression should be further explored, understanding that Twist1 and other associated downstream regulators could be exploitable in therapeutic designs for EMT→MET reversion, particularly at an early stage of tumour development.

Similar to the spatiotemporal signal programming in Twist1 expression in different metastatic phases, microRNAs equally regulate MET transition *via* down-regulation of many EMT-TFs such as ZEB1 and ZEB2 and Snail2 (Slug). In reverse, many EMT-TFs inhibit the transcription of microRNAs. In addition, microRNAs function in concert with the p53 to modulate the EMT pathway. Importantly, some molecular interactions involving the inactivation of p53 have connections with the downregulation of microRNAs. Inquisitively, the interconnections of p53, microRNA transcriptions, epigenetic mutation, and other associated factors remain an enticing area for further exploration as a possible target for induction of the EMT→MET pathway and potential therapeutic manipulations.

### Inhibiting the EMT Initiation

Inhibition of EMT in the early steps of invasion and metastasis, which perhaps promises to be the most cost-effective approach, has been the talking point in the scientific community of late. As earlier highlighted, the interplay and crosstalk between TFs and other signalling molecules orchestrate and mandate adjacent epithelial tumour cells to undergo EMT, leading to tumour progression, invasiveness, metastasis, and therapeutic resistance in some cancers. These TFs act as downstream effector molecules for the cascade. Thus, breaking the crosstalk between TFs and these pathways is a reasonable option for arresting tumour cells from metastasising and limiting drug resistance. Although these factors were for a long time considered “undruggable” targets (156), a deeper understanding of their specific expression pattern, degradation, mode of binding, protein/protein interaction, as well as interaction with other signalling pathways has changed this narrative. Additionally, it has also opened new possibilities to affect transcription factors as therapeutic targets for cancer treatment. Furthering the exploits through another perspective, signalling crosstalk, understanding that molecular modulators in one pathway are enzymatic (transcriptional) targets of another that sometimes compete with the original modulators, the undruggable bottleneck could end in a breakthrough.

Among the promising opportunities to indirectly or directly target a transcription factor are inhibition (or activation) at the expression level; inhibition through physical degradation; inhibition (or activation) at the protein interaction level; and inhibition (or activation) through the binding of a ligand-based molecule in an activation/inhibition pocket and inhibition (or activation) at the protein/DNA binding level. For example, when Snail was blocked in a high-grade serous ovarian cancer cell line (HGSOC), cancer stemness was reversed by decreasing CSC markers (CD117 and CD133) (157).

Similarly, Zhang et al. demonstrated that Snail knockout in MCF-7 breast cancer cells initially resistant to 5-FU resulted in reversal of EMT declined cell invasion and improved sensitivity to 5-FU (158). Consequent to the various studies about Snail and the correlation with chemoresistance and *cancer* stem cells, targeting Snail is a fascinating approach to overcome EMT and cancer resistance, which suggests that Snail inhibitors could prevent tumour recurrence. Inactivation of Twist1 expression by linolenic acid (ALA) treatment markedly reduces cell migration (159). Recently, a small molecule, CYD19, was identified to bind strongly with Snail and inhibited its interaction with CREB-binding protein (160). The altered interaction leads to impairment of CBP/p300-mediated Snail acetylation and rapid degeneration of Snail *via* the proteasomal pathway.

Furthermore, studies have shown that inhibition of γ-secretase would prevent the cleavage of the Notch receptor, thereby blocking Notch signal transduction (161, 162). In this way, the expression of Snail and ZEB proteins are kept in check so that E-cadherin retains its function to maintain epithelial characteristics. Thus, γ-secretase inhibitors can be clinically tried for treating human malignancies. Disappointingly, however, despite the repeated promising preclinical results on the control of tumour progression, gamma-secretase inhibitors (GSIs) have failed to demonstrate clinical benefit in most solid tumours. The poor clinical performance to date calls for important questions that are yet to be answered. Perhaps, refinement of tumour Notch expression profiles and further mechanistic understanding of GSIs will necessarily assist appropriate patient selection.

Also, more innovative approaches may include the rational design of combinatorial strategies to maximise the potential of these agents by sensitising tumours to traditional chemotherapeutics while also compromising tumour ability to engage treatment resistance programs. However, it is worthy of note here that caution should be applied while designing GSIs to eliminate undesired toxicity associated with the γ-secretase inhibitors, as they are involved in a wide array of cellular functions.

Even though, as earlier suggested, interfering EMT transition might be a step forward in the prevention of cancer metastasis, intratumour heterogeneity poses several hurdles against successful therapeutic design. As a result, understanding the E/M cellular transitional plasticity along a phenotypic spectrum would be central to more precise diagnoses and increasing the susceptibility of cancer cells to therapeutic manipulations, thus overcoming the current spate of drug resistance.

### Prospect of Single-Cell Analysis in the Composite Milieu of EMT

Several studies dispute the role of EMT in cancer metastasis as a result of their failure to detect EMT in the process, thus suggesting a striking controversy in cancer biology. Worse still, the use of individual EMT biomarkers from the bulk-tissue approach has repeatedly failed to answer the question of where gene upregulation or downregulation arises among the heterogeneous components involving malignant cells, the surrounding stroma and immune infiltrate. Thus, a method that can offer a direct and unbiased characterisation of individual cell’s (gene) expression to unveil intratumoural heterogeneity would therefore provide clarity over the age-old questions, and perhaps, paves way for targeted therapy. Therefore, the roles of novel technologies such as single-cell analysis would be invaluable to improve understanding of the underlying molecular mechanisms of tumour heterogeneity and investigation of diverse intermediates of E/M cells during EMT.

Intriguingly, it is now widely established that trans-differentiation of E/M cells and their intermediates are the main cause of intratumour heterogeneity and that the disseminated cancer cells need to revert towards a more epithelial phenotype to increase the sensitivity of neoplastic cells to therapeutic interventions. To therapeutically target EMT and indeed, the metastable pEMT state, it is essential to extensively evaluate the duplex modulations that shuttle cells towards or away from E and M states. Presumably, the bidirectional inhibitions that targets both EMT and MET might be an effective strategy to destabilise the M or hybrid states, while avoiding an escape route to a different resistant state. With this in mind, we anticipate the next few decades will represent a major acceleration in our knowledge of EMT and its entire spectrum of states. This is expected to aid in the stratification of patients into low- and high-risk cohorts while opening up entirely new avenues in the treatment of epithelial tumours.

Given the considerable heterogeneity of the tumour ecosystem, it is essential to analyse the molecular and genetic characteristics of each cell (163), rather than employing the bulk-tissue approaches. Fortunately, the emergence of single-cell sequencing technology has come to the rescue, enabling cells undergoing a transition to be inspected as single cell instead of collective cell populations. To simply put, with single-cell analysis, it would be possible to obtain complete genomic information of the entire cells concerning EMT gene regulatory circuits along the EMT continuum and compare them with the corresponding primary tumours. Through this, several clinically relevant genomic alterations could be discovered to provide valuable information for promising diagnostic and therapeutic opportunities. Specifically, single-cell RNA sequencing (ScRNA-seq), which provides high-throughput and high-resolution transcriptomic analyses of individual cells has been used to eliminate much of the background noise within a mixed population of cells. With a temporal profile, it will allow investigators to probe the genomic, transcriptomic, epigenomic, or other multiomics dynamics of heterogeneous cell populations because it can measure the distribution of genes and gene products (mRNA and proteins) from individual cells. With scRNA, one can encapsulate cell trajectories and developmental processes such as EMT by applying a scRNA-seq time course to assemble a cell trajectory map (164). Generating an EMT time-course to capture transient cell states at single-cell resolution informs the investigator with information on how this dynamic process occurs over time, thereby providing an elegant resource that is not available in any other known way. ScRNA-seq method is a recently developed technology with a promising prospect at providing a transcriptomic analysis of individual cells to address the inherent complexity of EMT milieu in tumour metastasis and its tumour environment. Despite the promising prospect of this novel method, some technical barriers have been identified.

Firstly, both cellular integrity and viability largely determines the success or otherwise of the subsequent single-cell analyses. It means that distinct cells need to be isolated from each other promptly while avoiding mechanical damage to the cells during the single-cell isolation process. However, this cellular segregation may impair cell integrity and enzymatic treatment using trypsin, collagenase, and/or papain to isolate single cells from tissues. In other words, it may affect cell viability or alter the transcriptional products. Hence, this necessitates the need for improve approaches to enable the efficient “gentle” extraction and capture of living cells and avoid the potential damage to single cells caused by enzymatic treatment.

Again, researchers have used scRNA-seq to analyse the association between therapeutic response and specific infiltrated immune cells in the tumour environment (165). At this point, another gap to be filled by ScRNA-seq is to enable rationale strategies that can identify drug-resistant cell populations associated with poor prognosis to achieve long-term treatment efficacy and combinatorial therapeutics that can aid in cotargeting multiple activated pathways.

## Conclusion

There is overwhelming evidence that the dynamic EMT program, which oversees the transformation of polarised epithelial cells to a mesenchymal phenotype in embryogenesis, is also operational in the development of the secondary metastatic niche. Hitherto, the molecular event is understood as a consequent disruption of the tight regulation in morphogenesis, where transcription factors play significant roles. Unfortunately, the EMT program, primarily driven by transcriptional repressors, is not only involved in tumour metastasis but has now been discovered as a molecular driver in drug resistance. To date, the problems of drug resistance and pharmacological limitation of tumour targeted therapy remain a significant challenge in cancer control interventions. Meanwhile, in recent times, several EMT transcriptional repressors have been inculpated in the metastatic cascade. As research begins to gain momentum, it is becoming increasingly clear that these factors would play considerable roles in targeted therapeutic strategies in the fight against cancer metastasis as well as cancer resistance to therapies. Thus, while it is currently clear that the EMT program is beyond the binary switch concept, we believe that a deeper understanding of the emerging roles of cellular plasticity which adapts tumour cell phenotypes through a metastable spectrum would also require a paradigm shift in investigational approach to upscale the common bulk-tissue methods to novel techniques such as single-cell analysis to address the lingering challenges in cancer management.

## Author Contributions

SI conceptualised and designed the study. KA designed the images. Both SI and KA developed the first draft, refined, edited, and revised the manuscript. IB formatted the references and contributed to the final editing. AM, MM, TO, and MO contributed to the content materials and final editing. All the authors contributed to the article and approved the submitted version.

## Conflict of Interest

The authors declare that the research was conducted in the absence of any commercial or financial relationships that could be construed as a potential conflict of interest.

## Publisher’s Note

All claims expressed in this article are solely those of the authors and do not necessarily represent those of their affiliated organizations, or those of the publisher, the editors and the reviewers. Any product that may be evaluated in this article, or claim that may be made by its manufacturer, is not guaranteed or endorsed by the publisher.

## Abbreviations

bHLH, basic helix-loop-helix; CSC, cancer stem cell; CNS, central nervous system; CTC, circulating tumour cell; cDNA, complementary deoxyribonucleic acid; DNA, deoxyribonucleic acid; EGF, epidermal growth factor; EMT, epithelial-mesenchymal transition; ER, oestrogen receptor; FGF, fibroblast growth factor; FGFR, fibroblast growth factor receptor; FOX, Forkhead box; FOXC2, Forkhead box C2; GSI, gamma-secretase inhibitor; GSC, goosecoid; GRHL2, grainy head-like 2; HTH, helix-turn-helix; HDAC3, histone deacetylase 3; HIF-1α, hypoxia-inducible factor-1 alpha; KLF8, Krüppel-like factor 8; LOX, lysyl oxidase; MMP, matrix metalloproteases; MET, mesenchymal-epithelial transition; MTA3, metastasis-associated protein 3; MAPK, mitogen-activated protein kinase; NICD, Notch intracellular domain; NES, nuclear export sequence; NF-KB, nuclear factor-KB; NCoR, nuclear receptor corepressor; PRRX1, paired-related homeobox 1; PI3K, phosphatidylinositol 3-kinase; PDGF, platelet-derived growth factor; PDGFR, platelet-derived growth factor receptor; RNA, ribonucleic acid; SRD, serine-rich domain; shRNA, short hairpin ribonucleic acid; siRNA, small interfering ribonucleic acid; TF, transcription factor; TGF-β, tumour growth factor-β; TGF-βR, tumour growth factor-β receptor; ZEB, zinc-finger E-box binding; scRNA-seq, single-cell RNA sequencing; pEMT, partial epithelial-mesenchymal transition.
